# Genome-wide association study of musical beat synchronization demonstrates high polygenicity

**DOI:** 10.1038/s41562-022-01359-x

**Published:** 2022-06-16

**Authors:** Maria Niarchou, Daniel E. Gustavson, J. Fah Sathirapongsasuti, Manuel Anglada-Tort, Else Eising, Eamonn Bell, Evonne McArthur, Peter Straub, 23andMe Research Team, Stella Aslibekyan, Adam Auton, Robert K. Bell, Katarzyna Bryc, Sarah K. Clark, Sarah L. Elson, Kipper Fletez-Brant, Pierre Fontanillas, Nicholas A. Furlotte, Pooja M. Gandhi, Karl Heilbron, Barry Hicks, Karen E. Huber, Ethan M. Jewett, Yunxuan Jiang, Aaron Kleinman, Keng-Han Lin, Nadia K. Litterman, Jey C. McCreight, Matthew H. McIntyre, Kimberly F. McManus, Joanna L. Mountain, Sahar V. Mozaffari, Priyanka Nandakumar, Elizabeth S. Noblin, Carrie A. M. Northover, Jared O’Connell, Steven J. Pitts, G. David Poznik, Anjali J. Shastri, Janie F. Shelton, Suyash Shringarpure, Chao Tian, Joyce Y. Tung, Robert J. Tunney, Vladimir Vacic, Xin Wang, J. Devin McAuley, John A. Capra, Fredrik Ullén, Nicole Creanza, Miriam A. Mosing, David A. Hinds, Lea K. Davis, Nori Jacoby, Reyna L. Gordon

**Affiliations:** 1 Vanderbilt Genetics Institute, Vanderbilt University Medical Center, Nashville, TN USA; 2 Division of Genetic Medicine, Department of Medicine, Vanderbilt University Medical Center, Nashville, TN USA; 3 23andMe, Inc, Sunnyvale, CA USA; 4 Computational Auditory Perception Group, Max Planck Institute for Empirical Aesthetics, Frankfurt am Main, Germany; 5 Department of Language and Genetics, Max Planck Institute for Psycholinguistics, Nijmegen, Netherlands; 6 Department of Music, Columbia University, New York, NY USA; 7 Department of Computer Science, Durham University, Durham, UK; 8 Department of Psychology, Michigan State University, East Lansing, MI USA; 9 Bakar Computational Health Sciences Institute, University of California, San Francisco, CA USA; 10 Department of Epidemiology & Biostatistics, University of California, San Francisco, CA USA; 11 Department of Neuroscience, Karolinska Institutet, Solna, Sweden; 12 Department of Cognitive Neuropsychology, Max Planck Institute for Empirical Aesthetics, Frankfurt am Main, Germany; 13 Department of Biological Sciences, Vanderbilt University, Nashville, TN USA; 14 Evolutionary Studies Initiative, Vanderbilt University, Nashville, TN USA; 15 Melbourne School of Psychological Sciences, University of Melbourne, Melbourne, Victoria Australia; 16 Department of Biomedical Informatics, Vanderbilt University Medical Center, Nashville, TN USA; 17 Department of Psychiatry and Behavioral Sciences, Vanderbilt University Medical Center, Nashville, TN USA; 18 Department of Molecular Physiology and Biophysics, Vanderbilt University, Nashville, TN USA; 19 Department of Otolaryngology—Head & Neck Surgery, Vanderbilt University Medical Center, Nashville, TN USA; 20 Department of Psychology, Vanderbilt University, Nashville, TN USA; 21 Vanderbilt Brain Institute, Vanderbilt University, Nashville, TN USA

**Keywords:** Genome-wide association studies, Human behaviour, Cognitive neuroscience, Human behaviour, Evolutionary genetics

## Abstract

Moving in synchrony to the beat is a fundamental component of musicality. Here we conducted a genome-wide association study to identify common genetic variants associated with beat synchronization in 606,825 individuals. Beat synchronization exhibited a highly polygenic architecture, with 69 loci reaching genome-wide significance (*P* < 5 × 10^−8^) and single-nucleotide-polymorphism-based heritability (on the liability scale) of 13%–16%. Heritability was enriched for genes expressed in brain tissues and for fetal and adult brain-specific gene regulatory elements, underscoring the role of central-nervous-system-expressed genes linked to the genetic basis of the trait. We performed validations of the self-report phenotype (through separate experiments) and of the genome-wide association study (polygenic scores for beat synchronization were associated with patients algorithmically classified as musicians in medical records of a separate biobank). Genetic correlations with breathing function, motor function, processing speed and chronotype suggest shared genetic architecture with beat synchronization and provide avenues for new phenotypic and genetic explorations.

## Abstract (web summary)

Niarchou et al. identify 69 genomic loci associated with people’s synchronization to a musical beat. The genetic architecture of beat synchronization was enriched for genes involved in early brain development and lifelong brain function.

## Main

Our tendency to perceive, create and appreciate rhythms in a variety of contexts (for example, speech, music and movement) is a key feature of the human experience^1–3^. Rhythmic patterns provide predictable and robust sensorimotor structure to everyday interactions^4,5^, helping guide our attention to communicatively important moments in time^6,7^. Even young children are sensitive to the social and linguistic signals carried by rhythm^8–10^, and parents use rhythmic vocalizations and synchronous movement (for example, lullabies and rocking) to interact with their infants from birth^11,12^. Rhythmic musical interactions are structured around the percept of a stable periodic pulse (termed the ‘beat’ in Western music and present in the music of most cultures^1,13^, though its precise instantiation in musical structure varies cross-culturally^14,15^). While music in general and rhythmic structures in particular vary globally^15–17^, there is evidence that the hierarchical beat structure of most music is robust to cultural transmission^2^ and indeed common in many types of music^1^.

Beat perception and synchronization (that is, perceiving, predicting and moving predictively in synchrony to a musical beat^18^) are important features of musical experiences across many human cultures and musical genres^1,19^. The predictive temporal mechanisms afforded by beat structure enhance general perceptual and learning processes in music, including melody perception and production, singing, and joint music-making^3,6^. While some features of rhythm perception and production vary across listeners from different cultures^13,19–21^, the same studies showed considerable consistencies across cultures in other features (for example, preference for beat-based isochrony). Musicality (broadly encompassing musical behaviour, music engagement and musical skill^22^) impacts society by supporting pro-social behaviour^11,23^ and well-being^24^. Many have proposed that beat perception and synchronization evolved in humans to support communication and group cohesion^18,22,25,26^. In modern humans, beat perception and synchronization are predictive of language and literacy skills^27,28^ and are related to cognition, motor function and social coordination^29^. The biology of beat synchronization thus has general importance for understanding the human ability to perceive and predict natural rhythms; may have relevance for characterizing phenotypes such as developmental speech–language disorders, which demonstrate associations with atypical rhythm^30^; and may further elucidate mechanisms of rhythm-based rehabilitation (for example, for stroke and Parkinson’s^31^).

Neuroimaging findings highlight auditory–motor networks in the brain underlying beat perception and production^32^, during which there is precise entrainment of neural oscillatory activity to musical signals, primarily involving motor planning areas and auditory regions of the brain, even during passive listening to music^33,34^. Neural mechanisms of entrainment, prediction and reward work in concert to coordinate the timing of beat-related expectancies to musical signals during listening, playing, singing and dance^26,34^. The substantial interindividual variation of beat synchronization^35^ is thought to be influenced, in part, by common genetic variation; thus, genetic approaches can be used to gain a foothold on the biological basis of musicality and human rhythm traits.

Indeed, twin-modelling and other family-based studies point to moderate heritability of rhythm-related traits such as duration discrimination^36,37^, rhythm discrimination^38^, isochronous sensorimotor production^39^ and off-beat detection^40^. Much less is known at the molecular level about human genome variation underlying rhythm and musicality more generally^41^, which to date has been investigated in relatively small samples^37^ due to the challenge of assessing such phenotypes in samples large enough to provide sufficient power to detect common variants with small effects (as expected for complex traits^42^). Large-scale genome-wide association studies (GWASs) of rhythm-based traits (for example, beat synchronization) are thus needed to advance this field. Our understanding of the biological underpinnings of beat synchronization, from its genetic architecture to its neural instantiation, behavioural manifestation and relationship to health, requires mechanistic multi-methodological approaches. Post-GWAS approaches (that is, enrichment of gene expression in central nervous system tissues and genetic correlations) can be deployed to illuminate the relationship between the genetic and neural architecture of music-related traits as well as shared underlying biology with other health traits.

Here we report a large-scale genome-wide interrogation of beat synchronization. Our approach was as follows (Supplementary Fig. 1). (1) We validated a subjective self-reported beat synchronization item (‘Can you clap in time with a musical beat?’, referred to in this paper as the ‘target question’), in relation to measured beat synchronization and rhythm perception task performance. (2) We performed a GWAS in *N* = 606,825 people to identify genomic loci associated with beat synchronization. (3) We further investigated the genetic architecture of beat synchronization by estimating single nucleotide polymorphism (SNP)-based heritability and partitioned heritability, and conducting gene property and gene-set enrichment analyses. We also evaluated the contribution of genomic regions that have experienced notable human-specific evolutionary shifts. (4) We validated the GWAS results by testing whether a cumulative sum of the genetic effects for beat synchronization detected in our GWAS (that is, polygenic score (PGS)) was significantly associated with algorithmically identified musical engagement in a separate sample. (5) We explored shared genetic effects (pleiotropy) on beat synchronization and other traits through genetic correlation and genomic structural equation analyses.

## Results

### Overview: validations of self-reported beat synchronization

**Fig. 1:**
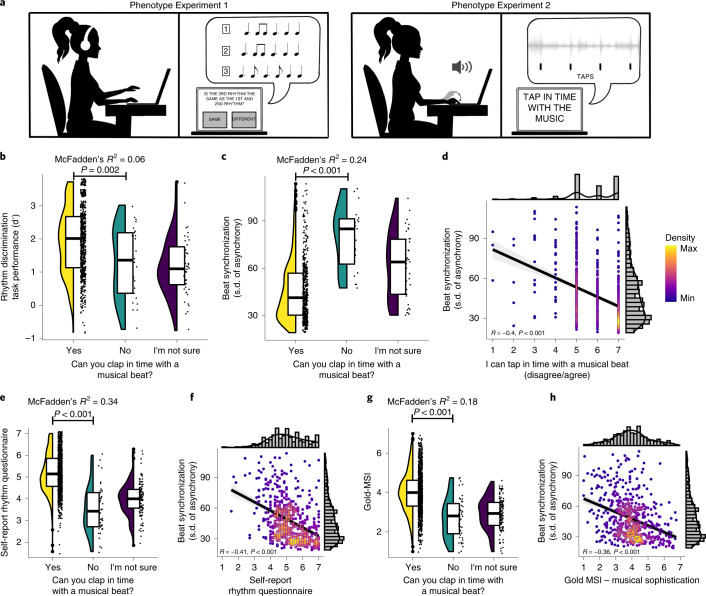
Overview and results of the phenotype validation studies. **a**, Schema of internet-based phenotype validation studies. In Phenotype Experiment 1, the participants performed a musical rhythm perception test and provided a self-report of the same target question in the GWAS study (‘Can you clap in time with a musical beat?’). In Phenotype Experiment 2, the participants performed beat synchronization tasks (which involved tapping to the beat of musical excerpts) as well as responding to the same target question, in addition to a series of other questionnaires about their musical engagement/ability and health traits. Illustration: Navya Thakkar. **b**, Phenotype Experiment 1 results (*N* = 724) show that rhythm perception task performance is correlated with Yes versus No responses to the GWAS target question (OR = 1.92, McFadden’s *R*^2^ = 0.06, *P* = 0.002). **c**–**h**, Phenotype Experiment 2 results. Beat synchronization task performance (*N* = 542) is highly correlated with Yes versus No responses to the target question (OR = 0.28, McFadden’s *R*^2^ = 0.24, *P* < 0.001); note that lower values of s.d. of the asynchrony correspond to more accurate tapping in time to the musical beat (**c**). Beat synchronization task performance is correlated with responses to a similar self-report question asked on a Likert scale (*N* = 542, *r* = –0.40, *P* < 0.001) (**d**). The self-reported rhythm questionnaire (seven-item scale, *N* = 1,412) is correlated with responses to the target question (McFadden’s *R*^2^ = 0.34, *P* < 0.001) (**e**). Beat synchronization task performance is correlated with the self-reported rhythm questionnaire (*N* = 542, *r* = 0.41, *P* < 0.001) (**f**). Goldsmiths Musical Sophistication Index (Gold-MSI) (a self-reported musical sophistication questionnaire) is correlated with responses to the target question (*N* = 1,412, OR = 4.16, McFadden’s *R*^2^ = 0.18, *P* < 0.001) (**g**). Beat synchronization task performance is correlated with Gold-MSI (*N* = 542, *r* = −0.36, *P* < 0.001) (**h**). Within each plot for **b**, **c**, **e** and **g**, distributions are displayed using violin plots (mirrored density plots showing probability density on the left), jittered individual data plots (right) and box plots in the centre (the horizontal line is at the median, the lower and upper edges correspond to the first and third quartiles, and the upper and lower whiskers extend from the edges to the value no further than 1.5× the interquartile range from the edge; data beyond the ends of the whiskers are called ‘outlying’ points and are plotted individually). In **d**, **f** and **h**, scatterplots are shown with dots coloured by density to illustrate distribution; the diagonal lines in the scatterplots represent regression lines with 95% CIs (shaded grey areas). All tests are two-tailed. Taken together, these results show that self-reported beat synchronization is a reasonable proxy of the trait. Source data

In light of prior work suggesting that musicality and rhythm skills are complex traits that can be quantified with both objective (experiment-derived) assessment and subjective self-reported data^43,44^, we performed a series of validations of the GWAS target question (that is, the self-report ‘Can you clap in time with a musical beat?’), in relation to rhythm perception and beat production tasks. Both studies were administered in English for consistency. We also explored the relationship between task-based beat synchronization ability, a self-reported rhythm scale and musicality. Study overviews and key results are summarized in Fig. 1.

#### Phenotype Experiment 1: rhythm perception task performance

In this experiment, *N* = 724 people (see Table 1 for the demographics) were asked the target question and performed a musical rhythm perception test (Supplementary Fig. 2). In each of the 32 trials of the task, the participants judged whether two rhythms were the same or different (Fig. 1a), following a standard procedure for assessing musical perception ability^45^ and utilizing rhythm sequences with simple (highly metrical) and complex (syncopated) rhythms^46^. The rhythm perception task yielded quantitative scores (*d*′). Individuals with better performance in the rhythm perception test (higher total *d*′) were more likely to answer Yes (versus No) to the target question (odds ratio (OR), 1.92; *P* = 0.002; McFadden’s *R*^2^ = 0.06; 95% confidence interval (CI), (1.27, 2.95); Fig. 1b). All tests in both phenotype experiments were two-tailed.

**Table 1:** Sample demographics for each of the three study samples (GWAS and Phenotype Experiments 1 and 2)

GWAS sample by phenotype group (response to target question)			
	Cases (Yes)	Controls (No)	
Total	555,660	51,165	
Males	226,188	23,998	
Females	329,472	27,167	
18 to 30 years old	57,898	5,186	
30 to 45 years old	135,168	12,909	
45 to 60 years old	150,939	13,312	
60 years old and over	211,655	19,758	
**Phenotype Experiment 1 (rhythm perception)**			
	*N*	Mean age in years	S.d. age
Total*	724	36.08	10.90
Males	387	34.95	10.60
Females	333	37.49	11.07
*Of the total *N* = 724; *N* = 3 did not report their age; *N* = 721 reported full demographics.			
**Phenotype Experiment 2 (beat synchronization and cross-trait phenotypic replication)**			
Full sample (questionnaires)	*N*	Mean age in years	S.d. Age
Total	1,412	36.34	11.93
Males	678	35.53	11.12
Females	728	37.15	12.61
Subset with valid tapping data	*N*	Mean age in years	S.d. Age
Total	542	35.24	11.39
Males	241	35.02	10.62
Females	300	35.43	12.00

#### Phenotype Experiment 2: beat synchronization task performance

We then validated self-reported beat synchronization phenotype (*N* = 1,412) as a proxy for directly measured beat synchronization ability. The participants (Table 1) completed a questionnaire on musicality, health and personality and were asked to tap in real time to the beat of four different musical excerpts (Supplementary Fig. 3). Beat synchronization tapping accuracy was assessed similarly to lab-based studies^35^, but with a recently developed online-based technology that precisely measures the asynchrony of participants’ taps along to music clips—that is, REPP (Rhythm ExPeriment Platform^47^). For additional details and preregistered hypotheses (H1–H6), see the Methods and Supplementary Methods and Results: section A. The key results of this study are summarized in Fig. 1 and Supplementary Table 1. Note that more accurate tapping is reflected in lower tapping asynchrony scores—that is, more accurate timing of taps in relation to the beat.

As predicted (Open Science Framework preregistered H1), individuals who responded Yes to the target question (‘Can you clap in time with a musical beat?’) had lower tapping asynchrony (OR = 0.28; *P* < 0.001; McFadden’s *R*^2^ = 0.24; 95% CI, (0.18, 0.42); Fig. 1c). Tapping asynchrony was also negatively correlated with responses to a highly similar item (‘I can tap in time to a musical beat’) when asked on a seven-point Likert agreement scale (1 = disagree; 7 = agree) (*r* = −0.40; *P* < 0.001; 95% CI, (−0.47, −0.33)) (H1a; Fig. 1d). Similarly, individuals with higher self-reported rhythmic ability (from another multi-item questionnaire) were much more likely to respond ‘Yes’ to the target question (OR = 7.34; *P* < 0.001; McFadden’s *R*^2^ = 0.34; 95% CI, (4.90, 11.52); Fig. 1e) and demonstrate lower tapping asynchrony (*r* = −0.41; *P* < 0.001; 95% CI, (−0.47, −0.33); Fig. 1f) (H2). Controlling for confidence judgements or confidence as a personality trait did not diminish the associations between self-report and tapping asynchrony (H3; Supplementary Methods and Results: section A). Musical sophistication^43^ was positively associated with the target question (OR = 4.16; *P* < 0.001; McFadden’s *R*^2^ = 0.18; 95% CI, (2.90, 6.12); Fig. 1g) and negatively correlated with tapping asynchrony (*r* = −0.36; *P* < 0.001; 95% CI, (−0.43, −0.28); Fig. 1h; H5). There was no credible evidence that musical sophistication or prior/current musician status interacted with the tapping asynchrony to predict responses to the target question (H6). All associations reported here were maintained when controlling for age, sex and education (Supplementary Table 1). Key analyses were repeated using vector length (the variability of the relative phase of participants’ tapping) as an outcome and showed the same pattern of results as s.d. of the asynchrony (Methods and Supplementary Methods and Results: section A). Taken together, these results show that the self-reported target question is a valid phenotype and that other similar self-reported rhythm measures are also valid proxies of beat synchronization.

### Beat synchronization GWAS

#### Genomic study population

The discovery GWAS sample consisted of *N* = 606,825 unrelated participants of European ancestry (see Table 1 for the demographics) who consented to participate in research with 23andMe, Inc. and answered Yes (91.57%) or No (8.43%) to the target question ‘Can you clap in time with a musical beat?’

#### GWAS results and SNP-based heritability estimation

**Fig. 2:**
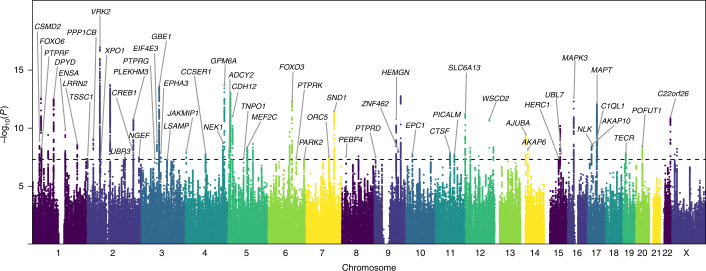
Manhattan plot of GWAS results of beat synchronization. Results of GWAS in *N* = 606,825 participants with 23andMe. The GWAS phenotype is the participants’ responses of Yes (*N* = 555,660) versus No (*N* = 51,165) to the question ‘Can you clap in time with a musical beat?’. The GWAS was performed with logistic regression, controlling for age, sex, the top five principal components for ancestry and genotype platform. The *x* axis shows chromosomal positions, and the *y* axis shows −log_10_ *P* values of the association between the alleles and the phenotype. Sixty-nine loci (70 sentinel SNPs, with one locus containing two independent sentinel SNPs) surpassed the threshold for genome-wide significance of *P* < 5 × 10^−8^ (dashed horizontal line). For illustration purposes, all SNPs with *P* < 5 × 10^−^^7^ are shown, and SNPs between *P* = 5 × 10^−^^7^ and 1 were downsampled to an evenly distributed draw of 30,880 SNPs; gene symbols for sentinel SNPs are notated when FUMA provided a gene mapped to the nearest sentinel SNP.

GWAS was conducted using logistic regression under an additive genetic model, while adjusting for age, sex, the first five principal components from genetic data and genotype platforms (Methods and Supplementary Methods and Results: section B). Seventy ‘sentinel’ SNPs (after two rounds of linkage disequilibrium (LD) pruning, first at *r*^2^ = 0.6 and then at *r*^2^ = 0.1, kb = 250) at 69 genomic loci reached genome-wide significance (*P* < 5 × 10^−8^; two-tailed; Fig. 2 (Manhattan plot), Table 2 (list of sentinel SNPs, their effect allele, minor allele frequency and *P* values) and Supplementary Table 2 (further details on the locus of each sentinel SNP)), with a total of 6,160 SNPs passing the genome-wide significance threshold. Sixty-seven loci were autosomal, and two were on the X chromosome; locus 28 contains two independent sentinel SNPs. A quantile–quantile plot is provided in Supplementary Fig. 4, and local association plots at each locus are provided in the data repository for the project (https://bitbucket.org/marianiarchou/beat-synchronization-gwas). The LD score regression (LDSC) intercept was 1.02 (s.e. = 0.01), and the ratio was 0.03, indicating that the majority of inflation in test statistics was due to true polygenicity instead of population stratification.

**Table 2:** Genomic loci and sentinel SNPs significantly associated with beat synchronization in the primary GWAS

Genomic locus	Sentinel SNP	Chromosome	Effect allele	Minor allele frequency	*P* value	Gene symbol
11	rs848293	2	G	0.42228	9.23 × 10^−18^	*VRK2*
26	rs62340585	4	G	0.20695	1.81 × 10^−14^	*GPM6A*
13	rs10168817	2	G	0.49299	1.94 × 10^−14^	NA
20	rs10779987	3	T	0.38175	2.21 × 10^−14^	*GBE1*
28	rs28392605	5	G	0.33904	8.93 × 10^−14^	NA
45	rs1832909	9	T	0.40687	1.78 × 10^−13^	NA
2	rs34762587	1	T	0.31379	2.25 × 10^−13^	*FOXO6*
60	rs7542	16	G	0.46184	2.41 × 10^−13^	*MAPK3*
5	rs10875125	1	C	0.15305	2.61 × 10^−13^	*DPYD*
35	rs9400241	6	C	0.28851	4.49 × 10^−13^	*FOXO3*
64	rs4792891	17	T	0.34013	7.07 × 10^−13^	*MAPT*
39	rs1468701	7	G	0.29172	3.62 × 10^−12^	*SND1*
50	rs10848650	12	G	0.42192	6.04 × 10^−12^	*SLC6A13*
29	rs2635634	5	T	0.45317	9.54 × 10^−12^	*CDH12*
67	rs9626920	22	G	0.41282	1.04 × 10^−11^	*MIRLET7BHG*
16	rs764299	2	G	0.26719	1.47 × 10^−11^	*PLEKHM3*
43	rs10984506	9	T	0.36558	1.66 × 10^−11^	*ANP32B*
53	rs1426371	12	G	0.25919	1.67 × 10^−11^	*WSCD2*
58	rs12913592	15	T	0.3596	6.13 × 10^−11^	NA
6	rs72700870	1	G	0.14377	1.42 × 10^−10^	*MCL1*
34	rs9388171	6	G	0.47595	2.16 × 10^−10^	NA
55	rs6572878	14	T	0.39477	3.48 × 10^−10^	*HAUS4*
4	rs11210206	1	T	0.31286	3.93 × 10^−10^	NA
28	rs72633496	5	T	0.43224	6.21 × 10^−10^	NA
10	rs7586405	2	G	0.30559	7.19 × 10^−10^	*PPP1CB*
63	rs3024293	17	T	0.23528	8.26 × 10^−10^	*C1QL1*
1	rs2061843	1	G	0.4001	1.19 × 10^−9^	*CSMD2*
19	rs1349028	3	T	0.25977	1.54 × 10^−9^	*EIF4E3*
25	rs4443239	4	T	0.2463	1.68 × 10^−9^	*C4orf27*
33	rs1901739	5	T	0.47772	2.14 × 10^−9^	NA
7	rs55678522	1	G	0.21629	2.25 × 10^−9^	*LRRN2*
61	rs8079923	17	T	0.25309	2.88 × 10^−^^9^	*AKAP10*
62	rs7501911	17	T	0.18191	3.34 × 10^−9^	*NLK*
66	rs6087848	20	G	0.44304	3.40 × 10^−9^	*POFUT1*
54	rs10744255	12	G	0.23229	4.24 × 10^−9^	NA
31	rs13163173	5	C	0.16597	4.51 × 10^−9^	*MEF2C*
3	rs2819333	1	T	0.37068	4.54 × 10^−9^	*PTPRF*
51	rs2453873	12	G	0.22254	5.17 × 10^−9^	NA
27	rs67264739	5	G	0.27395	5.54 × 10^−9^	*ADCY2*
69	rs4898322	X	T	0.14076	5.90 × 10^−9^	NA
56	rs2284901	14	G	0.37485	6.48 × 10^−9^	*AKAP6*
32	rs1596431	5	T	0.19182	7.42 × 10^−9^	NA
44	rs10978661	9	T	0.12006	7.74 × 10^−9^	*ZNF462*
23	rs4263335	4	G	0.49483	8.74 × 10^−9^	*JAKMIP1*
48	rs7939759	11	T	0.23981	1.23 × 10^−8^	*CTSF*
65	rs9710427	19	G	0.41536	1.32 × 10^−8^	*TECR*
21	rs12638746	3	G	0.33546	1.37 × 10^−8^	*EPHA3*
59	rs12909047	15	G	0.48251	1.49 × 10^−8^	*UBL7*
46	rs2505344	10	G	0.17674	1.51 × 10^−8^	*EPC1*
24	rs67816799	4	C	0.38188	1.56 × 10^−8^	*CCSER1*
15	rs10932201	2	G	0.46351	1.59 × 10^−8^	*CREB1*
49	rs526904	11	T	0.34865	1.60 × 10^−8^	*PICALM*
68	rs764935655	X	T	0.23454	1.83 × 10^−8^	NA
9	rs6548147	2	T	0.4402	2.05 × 10^−8^	*TSSC1*
52	rs10877461	12	G	0.29968	2.44 × 10^−8^	NA
41	rs11996434	8	G	0.27037	2.61 × 10^−8^	NA
40	rs1996148	8	G	0.31961	2.69 × 10^−8^	*PEBP4*
47	rs10885458	10	G	0.28314	2.69 × 10^−8^	NA
17	rs191373913	2	T	0.43899	2.74 × 10^−8^	*NGEF*
38	rs12056186	7	C	0.42875	2.93 × 10^−8^	*ORC5*
42	rs7856850	9	C	0.22184	3.07 × 10^−8^	*PTPRD*
36	rs13197257	6	T	0.27444	3.23 × 10^−8^	*PTPRK*
14	rs10497355	2	T	0.46078	3.43 × 10^−8^	*UBR3*
12	rs11692449	2	T	0.37522	3.45 × 10^−8^	*XPO1*
30	rs4704043	5	T	0.2827	3.65 × 10^−8^	*TNPO1*
18	rs43182	3	T	0.13443	3.80 × 10^−8^	*PTPRG*
57	rs62014217	15	G	0.20132	3.91 × 10^−8^	*HERC1*
8	rs476141	1	T	0.49868	4.49 × 10^−8^	NA
37	rs2849543	6	G	0.41591	4.60 × 10^−8^	*PARK2*
22	rs571760466	3	C	0.27511	4.81 × 10^−8^	*LSAMP*

Each locus has surpassed the genome-wide significance threshold (that is, *P* < 5 × 10^−8^); logistic regression was used to test associations in the GWAS. Further details (for example, chromosomal location) are provided in Supplementary Table 2.

The gene symbols are based on HUGO (HGNC) and appear in italics. These are all genes annotated to SNPs in *r*^2^ > 0.1 with the lead SNP; sentinel SNP in a given locus refers to an independent SNP from FUMA. The SNPs were mapped to genes on the basis of ANNOVAR annotation and being physically located inside a protein-coding gene using a 10-kb window. ‘NA’ indicates that the SNP is not within the 10-kb window of a gene. For presentation reasons, we included only one gene per SNP. For the full list of genes, see Supplementary Table 2.

The top-associated locus (rs848293) was mapped at chromosome 2 close to *VRK2 (*Vaccinia Serine/Threonine Kinase 2, which codes for a protein kinase with multiple spliced isoforms expressed in the brain) and *FANCL*, within a region previously linked to multiple neurological phenotypes^48,49^. Another strongly associated locus at chromosome 17 (rs4792891) included the Microtubule Associated Protein Tau (*MAPT*) gene, a locus associated with Parkinson’s disease^50^. The Mitogen-Activated Protein Kinase 3 (*MAPK3*) gene at 16p11.2, a region known to harbour rare variants that influence neurodevelopmental disorders^51^ and language-related phenotypes^52^, was also strongly implicated. We also identified a locus at Glycoprotein M6A (*GPM6A*), whose gene promoter contains a transcription factor binding site for *GATA2*, a gene previously related to music phenotypes^37^.

SNP-based heritability estimates on the liability scale^53^ ranged from 13% to 16% when adjusted for a range of estimated population prevalence for atypical beat synchronization (3.0% to 6.5%; Supplementary Table 3; see Supplementary Methods and Results: section C for an explanation of the prevalence estimates). The observed (unadjusted) genetic variance explained 5% (s.e. = 0.002) of the phenotypic variance.

#### Gene-based GWAS

Gene-based genome-wide association analyses performed with MAGMA yielded 129 genes surpassing the threshold of *P* < 2.56 × 10^−6^ (two-tailed; Supplementary Table 4), with the top two hits at *CCSER1*, in the 4q22 region in proximity to genes previously associated with multiple musicality phenotypes^54^, and *VRK2* (converging with the top locus in our SNP-based GWAS). Within these associations, we examined potential replication of 29 genetic associations with musicality in humans from prior reports^37,54,55^; none reached significance after genome-wide correction (Supplementary Table 5 and Supplementary Methods and Results: section D), either independently or as a gene set (*P* = 0.297).

### In silico functional analyses

#### Gene property and gene-set enrichment analyses

**Fig. 3:**
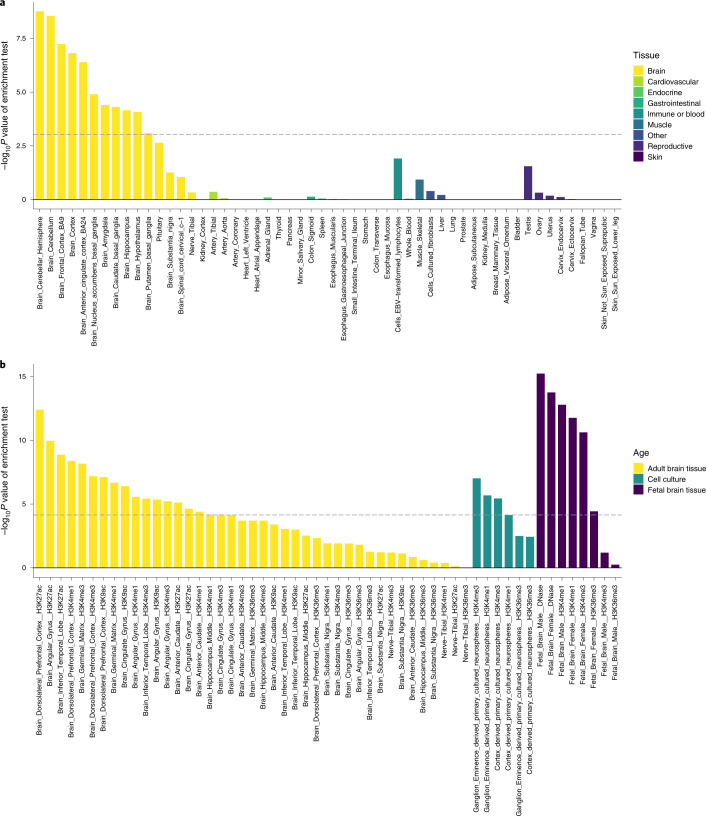
Genetic architecture of beat synchronization is enriched for brain-related expression. **a**, Genes associated with beat synchronization are enriched for expression in brain tissue. The results of MAGMA gene-property analysis are based on gene expression levels from GTEx v.8, in 54 tissues, conditioned on average expression across tissues. Associations with beat synchronization were significantly enriched in brain-expressed genes (−log_10_ *P* values are on the *y* axis, with tissue types on the *x* axis). The dashed line shows the *P* value threshold for significant enrichment after Bonferroni correction. **b**, Partitioned heritability shows enrichment in brain-specific regulatory regions of the genome. Partitioned heritability analysis was performed with LDSC–SEG. Tissue-specific regulatory elements are marked by histone 3 acetylation or DNase hypersensitivity (for open chromatin) and H3K4me1 (for enhancers). Regulatory regions in adult brain tissues are shown in yellow, regulatory elements in cell cultures are shown in teal and regulatory elements in fetal brain tissue are shown in dark purple. The graph shows −log_10_ *P* values on the *y* axis, with tissue and marker types on the *x* axis. The dashed line shows the *P* value threshold for significant enrichment after Bonferroni correction for the number of gene sets tested. Source data

To understand the biological functions and gene expression associations of beat synchronization, we performed gene-set analysis (GSA) and gene property enrichment analyses^56^ on the gene-based *P* values, using MAGMA^57^ implemented in FUMA^58^. The results of conditional gene property analysis (based on Genotype-Tissue Expression (GTEx) data tissue types^59^ and controlling for average expression) demonstrated that the genetic architecture of beat synchronization was significantly enriched in genes expressed in brain tissues (Fig. 3a), including cortex, cerebellum and basal ganglia (putamen, caudate and nucleus accumbens), converging with subcortical and cortical regions supporting beat perception and synchronization^34^.

To further examine potential biological functions associated with beat synchronization, we performed exploratory GSA^57^ (Supplementary Table 6). The genetic architecture of beat synchronization was enriched for two gene sets associated with nervous system function: gene sets for synaptic membrane adhesion (*P* = 1.01 × 10^−7^) and synaptic adhesion-like molecules (*P* = 8.35 × 10^−7^).

#### Partitioned heritability

Complementing these gene-based enrichment analyses, we also performed stratified LDSC^60^ on the GWAS results to partition heritability according to genomic properties, using specific functional categories to gain insight into the types of variation that contribute most to beat synchronization. Among broad SNP annotation categories^61^ (Supplementary Table 7), we found enrichment (all *P* < 9.6 × 10^−4^) of regions conserved in mammals (considered under purifying selection^62^) and regulatory regions marked by the acetylation of histone H3 at lysine 9 (H3K9ac, a marker for active chromatin) and the monomethylation of histone H3 at lysine 4 (H3K4me1, a marker for enhancers), supporting the hypothesis that the identified associations may affect gene regulation. We next used LDSC applied to specifically enriched genes (LDSC–SEG^63^) to determine whether genes expressed in specific cell or tissue types (conditional to the other annotations) are enriched for beat-synchronization-associated variants. For tissue-specific annotations of active chromatin and enhancers (marked by H3K9ac, H3K27ac, DNase hypersensitivity sites and H3K4me1), heritability was enriched in central-nervous-system- and skeletal-muscle-specific regulatory regions (Supplementary Table 8). Cell-type specific, multi-tissue chromatin and multi-tissue gene expression results are shown in Supplementary Figs 5–7, respectively. Enrichment in brain-specific regulatory elements, in multiple fetal and adult tissue-specific elements, and in central-nervous-system-specific cell cultures are shown in Fig. 3b.

### Evolutionary analyses

Given evolutionary hypotheses about the origins of rhythm^4,18,64^, we evaluated the contribution of regions of the human genome that have experienced significant human-specific shifts in evolutionary pressure, using stratified LDSC^53,60^. In particular, we analysed the contribution to beat synchronization heritability from variants in genomic loci that are conserved across non-human species but have elevated substitution rates on the human lineage^65^. Many of these human accelerated regions (HARs) play roles in human-enriched traits^66^, including cognition^67^. Two variants significantly (*P* < 5 × 10^−8^) associated with beat synchronization (rs14316 at locus 66 and rs1464791 at locus 20) fall within HARs. This is 11.2 times more overlap than expected by chance (*µ* = 0.178 overlaps; *P* = 0.017, based on 10,000 permutations). The rs1464791 variant is near *GBE1*, a gene associated with neuromuscular disease^68^, reaction time^69^ and cognitive impairment^70^. Applying LDSC to consider the full set of association statistics, we find that genetic variants in HARs contribute 2.26 times more to the observed heritability of beat synchronization than would be expected if heritability were distributed uniformly across variants (*P* = 0.14). Given the small number of common variants within HARs, this stratified heritability analysis is substantially underpowered (0.17% of variants considered are in HARs). The general agreement of these two approaches supports the enrichment of functional variation relevant to beat synchronization in HARs. We also evaluated the contribution of genetic variants detected in the Neanderthal genome to the heritability of beat synchronization (Supplementary Methods and Results: section E and Supplementary Table 9).

### PGSs for beat synchronization are related to musicality

We investigated whether the polygenic model of beat synchronization from the GWAS would differentiate self-identified musicians from non-musicians in a separate sample. Musicians (*N* = 1,259) and matched controls (*N* = 4,893) were drawn from a study^71^ that algorithmically identified musically active patients in a health-care research database (Methods and Supplementary Methods and Results: section F). PGSs for beat synchronization were significantly associated with musical engagement (OR = 1.34 per s.d. increase in PGS; *P* < 2 × 10^−16^; Nagelkerke’s *R*^2^ = 2%; 95% CI, (1.26, 1.43)), consistent with beat synchronization capturing a dimension of musicality.

### Cross-trait analyses

#### Genetic correlations

**Fig. 4:**
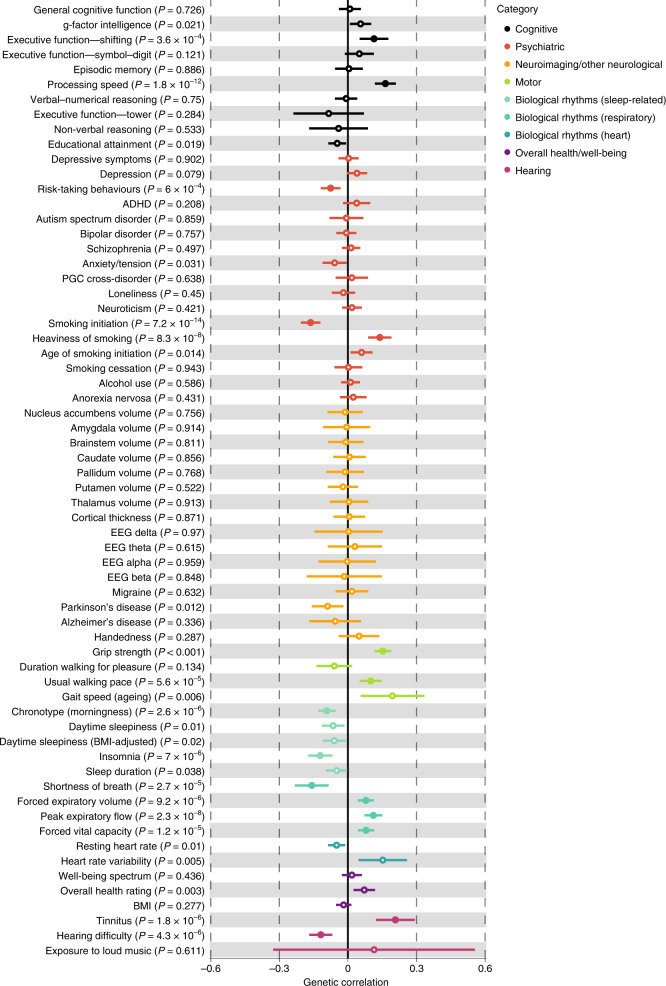
Cross-trait genetic correlations with beat synchronization. Results of exploratory genetic correlation analyses between beat synchronization and 64 traits from seven domains, conducted with LDSC. The *x* axis is magnitude of genetic correlation (*r*_g_) with standard error visualized, and the (uncorrected) *P* values for each trait’s correlation with beat synchronization are shown next to each trait label. Significant genetic correlations (two-tailed test; the significance threshold was set by adjusting for 64 comparisons with a threshold of *P* < 7.8 × 10^−4^) are shown with filled-in circles; empty circles are results that did not pass this threshold. See Supplementary Methods and Results: section G for details on the source studies. There are significant positive associations between beat synchronization and two of the cognitive domain GWASs, associations with smoking and risk-taking, two associations with hearing traits, two positive associations with motor function, and multiple associations with other biological rhythms (morning/evening chronotype, insomnia and four breathing-related traits). Source data

To determine whether beat synchronization shares genetic architecture with other traits, we tested genetic correlations^72^ between beat synchronization GWAS and available GWAS of 64 traits classified into seven domains (see Supplementary Table 10 and Supplementary Methods and Results: section G for the details). There were 15 statistically significant genetic correlations (*P* < 7.8 × 10^−4^) (Fig. 4 and Supplementary Table 11). The results included positive correlations with motor function (grip strength and usual walking pace) and heaviness of smoking, and negative correlations with risk-taking and smoking initiation. There were two correlations with hearing traits (a positive correlation with tinnitus and a negative correlation with hearing difficulty). From the cognitive traits, processing speed (faster perceptual motor speed) was genetically correlated with beat synchronization, in addition to executive function—shifting (from a GWAS of trail-making, a task that involves complex processing speed). There were multiple associations with other biological rhythms: breathing function traits (positive associations with peak expiratory flow, forced expiratory volume and forced vital capacity, and a negative correlation with shortness of breath) and sleep-related traits (negative associations with insomnia and morning chronotype).

#### Genomic structural equation modelling

**Fig. 5:**
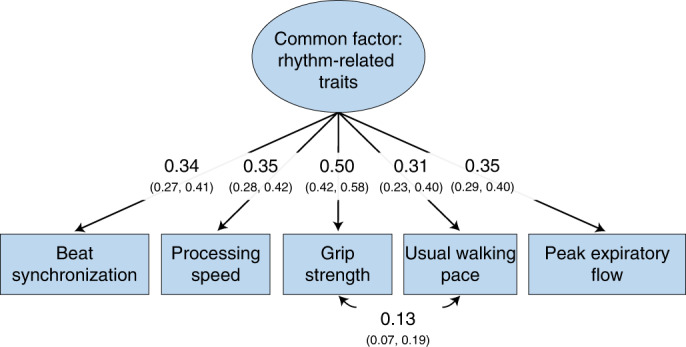
Genomic SEM model of beat synchronization and rhythm-related traits. The best-fitting genomic structural equation model of beat synchronization with GWASs of processing speed, grip strength, usual walking pace and peak expiratory flow. The 95% CIs of factor loadings and correlations are displayed in parentheses. The results suggest that beat synchronization was associated with the other traits through a set of common genetic influences. Model fit: *χ*^2^ (4) = 10.85, *P* = 0.028, comparative fit index (CFI) = 0.983, standardized root mean squared residual (SRMR) = 0.017. Source data

We conducted genomic structural equation modelling (SEM)^73^ to examine whether specific associations between beat synchronization and a subset of associated traits (for example, musculoskeletal strength, walking pace, breathing function and processing speed) that are known to be related among each other in prior research^74–76^ represent distinct genetic relationships or share a common set of genetic influences with beat synchronization. The best-fitting model, displayed in Fig. 5, included a common genetic factor that accounted for genetic correlations among beat synchronization, grip strength, processing speed, usual walking pace and expiratory flow. This common factor explained 11.6% of the total variance in the beat synchronization GWAS and 9.6–25.0% of the variance in the other GWASs (Supplementary Methods and Results: section I).

#### Common factor GWAS: rhythm-related traits

Using genomic SEM, we conducted a multivariate GWAS (Supplementary Methods and Results: section I) on the latent genetic factor from the model presented above and portrayed in Fig. 5. The heritability of this latent genetic factor was 7.27% (s.e. = 0.25%), and there were 130 genome-wide significant loci (Supplementary Table 12 and Supplementary Fig. 8). Heritability was enriched for genes expressed in cerebellum (Supplementary Fig. 9).

#### Cross-trait phenotypic extension of genetic correlations

Data from Phenotype Experiment 2 were analysed to examine whether a subset of genetic correlations would be reflected in true phenotypic associations (preregistered H4; Supplementary Methods and Results: section J). Less accurate beat synchronization was weakly associated with a morningness preference (*r* = −0.10, *P* = 0.015), more shortness of breath (*r* = −0.16, *P* < 0.001) and smoking 20 or more (lifetime) cigarettes (*r* = −0.11, *P* < 0.001) (Supplementary Table 13). In other words, accuracy in beat synchronization was correlated with eveningness chronotype, reduced shortness of breath when walking on level ground and smoking abstinence (these associations go in the same direction as the genetic correlations; moreover, these associations remained significant after controlling for age, sex and education, and/or removing professional musicians from the sample).

#### Additional sensitivity analyses

Our results are robust to several potential biases (Supplementary Methods and Results: sections K–M). The GWAS beat synchronization results are not driven by shared genetic effects with general cognitive ability or educational attainment (these traits were selected on the basis of prior associations between rhythm and cognition^39^ and, more broadly, between musicality and educational attainment^43^) or by subtle residual population substructure, and the *MAPT* association is not confounded with Parkinson’s disease.

## Discussion

Our GWAS revealed highly polygenic architecture of the human capacity to synchronize to a musical beat, representing a substantial advancement of our understanding of the genomic basis of a musicality phenotype. Heritability of beat synchronization is enriched for functions of the central nervous system on a number of dimensions: SNPs involved in neural development and brain-specific regulatory regions of the genome, genes involved in synaptic function, and gene expression in cortical and subcortical brain tissues aligned with auditory–motor regions previously linked beat perception and synchronization^34^. PGSs for beat synchronization were associated with self-identified musicians in a separate cohort, showing that the GWAS taps into the larger construct of musicality. Genetic correlations pointed to pleiotropy between beat synchronization and biological rhythms (including breathing function, walking pace and chronotype), paving the way to a better understanding of the biological underpinnings of musicality and its health relevance.

In a series of phenotypic experiments, we also demonstrate that self-reported beat synchronization/rhythm measures can be used in large-scale population-based studies as suitable proxies for measuring individual differences in beat synchronization. Our findings indicate that the GWAS phenotype beat synchronization question was highly related to beat synchronization task performance (that is, accuracy in tapping along to musical excerpts). Clearly, the self-report is an imperfect correlate of beat synchronization; nevertheless, we demonstrate that it is a suitable proxy for very large-scale studies in which task administration is impractical. Furthermore, the GWAS phenotype is also significantly associated with rhythm perception task performance^46^, a multi-item rhythm questionnaire and a well-established assessment of musical sophistication^43^. These results converge with earlier work showing shared variance among task performance of beat synchronization, rhythm perception and musical engagement/training^44,77–80^. The phenotypic associations were robust to demographic factors and self-confidence and were not driven by the presence of professional musicians in the sample. These phenotype validation studies represent critical groundwork^81^ enabling brief rhythm self-report questionnaires to be deployed online in large-scale population genetic cohorts.

With 69 loci surpassing the threshold for genome-wide significance, the polygenic architecture of the beat synchronization GWAS aligns with expectations for complex traits^82,83^. The top-associated locus mapped to *VRK2*, a gene previously associated with behavioural and psychiatric phenotypes (that is, depression^84^, schizophrenia^85^ and developmental delay^86^), suggesting a biological connection between beat synchronization and neurodevelopment. The SNP-based heritability of beat synchronization on the liability scale was moderate, ranging from 13% to 16%, similar to heritability estimates of other complex traits (for example, chronotype GWAS^87^) and consistent with moderate heritability estimates of musical rhythm abilities reported in twin studies^38–40^. Still, the limitation of the heritability adjustment on the liability scale is that the exact population prevalence of atypical beat synchronization is unknown and had to be estimated on the basis of other indices of rhythm (Supplementary Methods and Results: section C); this limitation should be addressed in future work.

We examined potential mechanisms linking genetic variation to neural architecture of the beat synchronization trait using multiple in silico enrichment methods. The results showed enrichment of the heritability of beat synchronization in many brain tissues including cerebellum, dorso-lateral prefrontal cortex, inferior temporal lobe and basal ganglia nuclei (that is, putamen, caudate and nucleus accumbens). This pattern of results probably reflects a genetic contribution to subcortical–cortical networks underlying musical rhythm perception and production^32,34^; furthermore, the enrichment of brain-tissue-specific enhancers and active-regulatory regions in tandem with gene expression enrichments in brain tissue suggests that regions of the genome involved in the regulation of gene expression within the beat perception and synchronization network contribute to phenotypic variance. Moreover, the partitioning heritability chromatin results showed an enrichment in both fetal and adult brain tissues, suggesting that beat synchronization may be the result of neurodevelopmental or basic brain processes. Gene-set enrichments were also observed for synaptic function in the nervous system. Taken together, these results are a building block towards understanding how genes influence neural processes during beat perception and production, complementing results obtained with neuroimaging methods^88–93^.

Insights about the evolution of rhythm traits are suggested by the occurrence of two of the beat-synchronization-associated loci in HARs of the genome. In particular, rs1464791 is an expression quantitative trait locus that regulates the expression of *GBE1* in multiple tissues, including muscle^59^; *GBE1* is also linked to neuromuscular disease^68^ and reaction time^69^. HARs are involved in many functions, so it is difficult to explicitly link their accelerated evolution to beat synchronization. It is too early to tell whether the overlap between beat-synchronization-associated loci and those two HARs supports evolutionary theories about music (for example, joint synchronous music-making has been posited to exert selective pressures in early humans by enhancing group social cohesion and family bonding^26,94^). The contribution of the genetic architecture of motor function to beat synchronization is further suggested by enriched heritability of SNPs that are enhancers located in musculoskeletal-tissue-specific regulatory regions of the genome, as well as our findings of genetic correlations between walking pace, musculoskeletal strength and beat synchronization.

Moreover, our findings are promising for future large-scale genomic interrogations using comprehensive music phenotyping yielding continuous musicality variables (whether questionnaire-based^43,95^ or measured aptitude-based variables^38^), providing a path to examine potential genetic correlations between beat synchronization and other musical traits, such as music training or pitch discrimination, in line with family-based findings^36–38,41^. While the current data show a clear connection between beat synchronization and broader musicality at the phenotypic and genetic levels, further genomic investigation in well-powered samples is needed to disentangle the specificity of genetic influences on beat synchronization from other genetic influences on musical traits, or motor or auditory function. Finally, although our GWAS was based on self-report, the magnitude of the sample size bolstered statistical power. This is important because previous GWASs of other health traits based on self-report have effectively replicated associations from other GWASs of deeper phenotypes^83^, and it is generally acknowledged that large sample sizes can overcome some of the limitations arising from modest measurement error^96^.

Moving in synchrony to a musical beat encompasses beat perception and extraction, motor periodicity, metre perception and auditory–motor entrainment^4,32,97^ (Glossary in the Supplementary Information). Despite this complexity, beat is a highly frequent feature of many musical systems^1,3,26^. Indeed, we found that the heritability of beat synchronization is enriched in auditory–motor regions known to be active during rhythm tasks^34^. It should be noted that beat perception and production do not depend on musical training or music genre, and atypical beat synchronization is not linked to lack of music exposure^98^. Although sensitivity analyses did not provide any evidence that population stratification affected our findings, the current study—along with all others conducted with currently available GWAS methods—has the potential to be affected by residual population stratification effects that cannot be completely ruled out^99^; this is a known problem in the field of human genetics that many groups are currently working to address. A limitation of the current work is also the restriction of the genetic sample to a European ancestry (due to GWAS methodology constraints); investigating beat synchronization, musicality and cross-trait correlations in populations of non-European ancestry should be a future priority for capturing the spectra of musicality traits in a wider range of ethnic, cultural and socio-economic contexts^100^. Guidelines for ethical and socially responsible conduct of research on the genetics of musicality are outlined in Box 1; for a more extensive discussion of these ethical and social issues, please see ref. ^101^.

We replicated previous findings implicating location 4q22.1 in musicality-related traits^36,55^ (*CCSER1* was the top-associated gene in our MAGMA analysis) but did not find support for previous gene associations from a set of genes that was drawn from prior candidate-gene, linkage and GWAS studies with relatively small samples^54^. This is potentially due to well-known methodological problems with these methods, particularly when applied to complex traits in small samples^102^. Without a second comparably sized GWAS available within which to conduct replication of the loci discovered in the primary GWAS, we were still able to demonstrate the generalizability of these results by showing that the PGS for beat synchronization predicts a musical trait in a separate biobank sample. The GWAS results of beat synchronization were nearly identical even after conditioning the results on GWASs of educational attainment and general cognition (g-factor); these results align with twin findings of specific genetic effects of rhythmic aptitude over and above any common genetic influences between rhythm and non-verbal cognitive variables^39,103^. Moreover, given the likely capturing of genetic variation related to socio-economic status^104^ by the educational attainment GWAS summary stats, as well as the observation that our beat synchronization GWAS loci are robust to educational attainment, socio-economic status is unlikely to play a major role in our findings.

Our cross-trait explorations revealed pleiotropic effects between beat synchronization and several breathing-related phenotypes (peak expiratory flow, forced vital capacity, forced expiratory volume and shortness of breath). We demonstrated phenotypically that more accurate beat synchronization task performance was related to a lower likelihood of shortness of breath, mirroring the genetic correlations between beat synchronization and breathing function. In light of our genetic correlation between beat synchronization and three categories of traits (breathing, motor and cognitive functions) previously shown to be genetically interrelated during the ageing process^74,75^, we used genomic SEM to uncover shared genetic variance among beat synchronization and enhanced breathing function, greater grip strength, faster walking pace and faster processing speed. Poor beat synchronization could be tied to certain health risks during ageing, in light of other genetic and epidemiological work showing that lung function decline predicts later declines in motor function and psychomotor speed in older adults^105–108^.

The genetic correlation results suggest that beat synchronization shares common biology with a constellation of health traits, converging with the growing literature on the overlapping biomechanical and perceptual mechanisms of rhythms harnessed during synchronization, communication, muscle tensioning and breathing; these relationships start very early in development^109,110^. The cerebellum in particular plays important roles in the control of coordinated movement, balance, respiration, dance and even rhythm perception during passive listening to music^33^. Indeed, our multivariate GWAS of rhythm-related traits demonstrated enriched heritability of genes expressed in cerebellar tissue, potentially of note in relation to experimental findings of functional synchronization of respiratory and upper limb movements during vocalization^5^. Moreover, ‘beat gestures’ in speech involve the cerebellum^111^ and are inextricably linked to respiration, upper limb movement and postural control, all of which may be biomechanically related to tapping or clapping to music.

Another dimension of biological chronometry was captured in the genetic correlation between chronotype and beat synchronization, which we replicated phenotypically (individuals who self-identified as ‘evening people’ tended to tap more accurately to music, even after removing professional musicians from the analysis). These results complement recent evidence of the increased prevalence of eveningness in musicians^112^, indicating that the relationship between chronotype and musicianship cannot be explained solely by environment (that is, the nocturnal job demands of professional musicians) and that other shared biological factors may play a role. Given the genetic correlation between beat synchronization and lowered incidence of insomnia, the relationship between regulation of sleep, musicality and rhythm represents an area for further exploration.

Our GWAS effectively identified alleles at 69 separate loci differentially associated with typical versus atypical beat synchronization, complementing existing evidence of underlying neural mechanisms^77,79,80,98^. Future genetic studies could study beat synchronization as a continuous trait, through either self-report or internet-based task paradigms (that is, REPP^47^). Prior literature on liability threshold models has shown that case–control GWASs of complex traits yield similar results to those obtained through continuous phenotypic measures (for example, the genetic architecture of continuous measures of psychiatric symptoms is highly similar to the genetic architecture of cases versus controls^113^). Moreover, the use here of a population-based control group that are not ‘super-controls’ (that is, controls screened not only for the condition studied but also for additional traits that may or may not be related to the condition^114^) increases the likelihood that the genetic correlations that we uncovered are reliable and not biased upwards^115^.

Taken together, our results advance knowledge of the biological basis of beat synchronization by identifying genomic regions associated with individual differences in beat synchronization, estimating its cumulative SNP-based heritability, successfully applying a PGS model in a separate genetic sample and exploring the enrichment of heritability in genes tied to central nervous system function. Movement in synchrony with a musical beat is a fundamental feature of music, and sensitivity to the beat emerges early in development, supporting childhood development in numerous ways^3,11,27,30^ and with importance over the lifespan^116^. We have elucidated the genetic architecture of beat synchronization and revealed its health relevance through cross-trait analyses. This study also provides a solid foundation for future exploration of how specific genetic variants contribute to neural mechanisms of entrainment, prediction and reward harnessed during musical interactions^117^.

Box 1 Initial ethical and social considerations related to the genetics of rhythm

1. **Genome-wide associations with beat synchronization are not deterministic**. The present results show that genetic influences accounted for only a small portion of human variation in beat synchronization. Environmental influences are the primary drivers of rhythmic accuracy. The value of this work arises not from the hypothetical ranking of interindividual differences in beat synchronization but from the discovery that variation in the human genome partly gives rise to the shared experience of musical rhythms. Genomic associations with beat synchronization cannot be used to make deterministic predictions about individual abilities or aptitudes^139,140^. The nuances of these results should be kept at the forefront of the interpretation of GWAS findings and are especially important during public dissemination.
2. **Historical context matters and lays the foundation for vigilant ethical and social conduct of research on musicality traits**. Early research on individual differences in musicality in the early 1900s was pursued not only using what we now recognize as highly culturally biased assessments but also explicitly through the lens of eugenics^141^, similar to early research on individual differences in cognition. We strongly condemn the intent and design of eugenic-focused studies. The historical context of cognitive genomics and how it intersected with racism and eugenics-motivated early music cognition studies heightens the present need for an ethical framework guiding responsible conduct and use of this research.
3. **Ethically and socially responsible conduct of research on the genetics of musicality includes conscious decision-making throughout the life cycle of research**. Decisions about what to study, how to study it, how to communicate results and how to apply results (for example, clinical translational or educational applications) should take place with consideration of historical context and responsible future applications.
4. **Inclusive and diverse studies of rhythm and other musicality traits should include populations of diverse ancestries**. A limitation of the current study is the restriction of the sample to only European ancestry for technical reasons (that is, the allele frequencies used to compute GWAS associations vary across ancestries). While genes carry out essentially the same functions in all humans, the specific variation that tags each gene differs as a consequence of population demography over the course of human evolution. Therefore, to fully understand the genetic architecture of a complex trait like beat synchronization, GWASs within other genetic ancestries should be pursued in the immediate future.
5. **PGSs and similar analyses applied to individual genetic data should not be used to make deterministic individual inferences or rankings about musicality**. For example, polygenic embryo selection^142^ for beat synchronization (or other musicality traits) is not scientifically justifiable or ethically sound. Likewise, it would be inadvisable and harmful to make decisions about cultural enrichment, such as access to music lessons, on the basis of PGSs.

Ethical and social issues related to the conduct of research on the genetics of musicality are discussed more extensively in ref. ^101^. In addition, a live FAQ for the current study is hosted at https://www.vumc.org/music-cognition-lab/FAQbeatGWAS.

## Methods

### Phenotype validation studies

#### Phenotype Experiment 1

##### Overview

Phenotype Experiment 1 was designed to determine whether self-reported rhythm abilities measured with the question used in the GWAS (that is, ‘Can you clap in time with a musical beat?’) would be associated with task-based rhythm perception performance. The study was conducted in Amazon’s Mechanical Turk and received ethical approval from the Columbia University Institutional Review Board; the participants gave their written informed consent, and the research complied with all relevant ethical regulations. We selected the Beat-Based Advantage paradigm as a rhythm discrimination (perception) test due to its design of stimuli with simple and complex metres^118^ and prior history investigating individual differences in rhythm perception in a variety of brain and behavioural studies in adults and children with typical and atypical development^46,119–121^ as well as feasibility for internet-based adaptation. A questionnaire (self-report questions) was administered before the perception task to avoid biasing participant self-report responses by how they perceived their own task performance. The participants were compensated about US$1.60–US$2.00 for participation; see Supplementary Methods and Results: section A for additional details on procedure, compensation and the self-report questionnaire. In both phenotypic experiments and in the GWAS, note that data collection and analysis were not performed blind to the conditions of the experiments.

##### Participants

The study sample was *N* = 724 participants recruited anonymously in Amazon’s Mechanical Turk. All consented and passed a common headphone check^122^ that guarantees good listening conditions and the ability to follow basic instructions; this test also effectively filters out bots. The participants (333 females, 387 males and 4 self-reported ‘other’) were 18–73 years old (mean, 36.1 years; s.d., 10.9) with 0–45 years of self-reported musical experience (mean, 3.7 years; s.d., 5.7), representing an average degree of musical experience (see the norms in ref. ^43^); the demographics are reported in Table 1 (note that *N* = 3 did not report their age). No exact sample size was predetermined, but the final *N* was in line with expectations for participation across two waves of recruitment via Mechanical Turk.

##### Rhythm perception task

The stimuli for the rhythm perception task consisted of 32 rhythms drawn from prior work^46,118^. For each participant, we randomized with probability of 0.5 the occurrence of ‘simple’ rhythms (with a strong beat-based metrical structure and generally easier to discriminate) and ‘complex’ rhythms (with a weaker metrical structure due to syncopation and generally more challenging to discriminate). Each rhythm was presented using pure tone stimuli in one of six frequencies (294, 353, 411, 470, 528 and 587 Hz, selected at random) and one of four durations (interstimulus interval of 220, 230, 240 and 250 ms). Each trial consisted of three rhythms separated by 1,500 ms of silence; there were 32 trials of the task. The two first presentations were always identical, and in half of the trials (counterbalanced), the third rhythm was also identical (standard condition); in the other half of the trials, the rhythm differed by having one interval swapped (deviant condition). The pairings and structure of standard and deviant trials were taken from ref. ^46^. The participants were instructed that in each trial, they would listen to the series of three rhythms (the first two were always identical, and the third could be the same or different), and they had to indicate whether the third rhythm was the same or different (Supplementary Fig. 2). Additional technical details are provided in the Supplementary Methods and Results: section A.

#### Data analysis

##### Self-report

Responses to the target question were as follows: *N* = 654 (90.3%) participants answered ‘Yes’, *N* = 25 (3.5%) answered ‘No’ and *N* = 45 (6.2%) answered ‘I’m not sure.’ Regarding an additional self-report question, ‘Do you have a good sense of rhythm?’, *N* = 503 (67%) answered ‘Yes’, 102 (14%) answered ‘No’ and *N* = 117 (16%) answered ‘I don’t know’. *N* = 488 answered ‘Yes’ to both questions; the tetrachoric correlation between these two self-report questions was *r* = 0.73.

##### Rhythm perception test

Responses to the rhythm perception test were analysed using signal detection theory^46,123^; this method is appropriate for discrimination tasks where the participant has to categorize stimuli along some dimension, with the resulting *d*′ values representing the strength of detection of the signal relative to noise. We calculated *d*′ values on the 32 test trials. As expected from prior work^46,124^, individuals performed better at discriminating simple rhythms (mean *d*′, 1.98; s.d., 0.91) than complex rhythms (mean *d*′, 1.43; s.d., 0.97) (*t*(724) = 11.11, *P* < 0.001, Cohen’s *d* = 0.58).

To examine whether the target question was related to the objective (experimenter-measured) performance on the rhythm perception test, we performed a logistic regression analysis in which the clap-beat target question (Yes versus No) was the outcome and quantitative scores on the rhythm perception test (*d*′ scores) were the predictor. Covariates included age, education and sex. McFadden’s *R*^2^ was also computed. We did not include ‘I’m not sure’ in the regressions, because this response was not available for data analysis in the GWAS. Given that the simple rhythms have a strong metrical structure that is known to facilitate detection and synchronization of the beat^46^, we also tested whether performance on the simple rhythm trials predicted self-reported beat synchronization (that is, those who responded Yes to the clap-beat question). All continuous measures met the assumption of normality (skew, ±2; kurtosis, ±4). See Supplementary Methods and Results: section A for additional analyses.

#### Phenotype Experiment 2

##### Overview

The aims of Phenotype Experiment 2 were twofold: (1) to validate self-reported beat synchronization phenotype as a proxy for objectively measured beat synchronization ability and (2) to explore phenotypic associations between rhythm/beat synchronization and assorted traits found to be genetically correlated with beat synchronization. Phenotype Experiment 2 was preregistered with the Open Science Framework (https://osf.io/exr2t) on 8 July 2020, prior to data collection. This internet-based study consisted of a beat synchronization task to assess the accuracy of participants’ tapping in time with musical excerpts, and a series of questionnaires assessing self-reported rhythm, musicality/music engagement, selected health traits, confidence as a personality trait and demographics. We used REPP^47^ to measure the participants’ tapping responses online with high temporal fidelity. The item from the GWAS study, ‘Can you clap in time with a musical beat?’ with possible responses Yes/No/I’m not sure, is referred to as the ‘target question.’

We tested the following hypotheses. First, self-report responses to the target question will be correlated with beat synchronization task performance (that is, the accuracy of tapping to the beat of music), such that individuals who respond Yes to the target question are predicted to tap more accurately to the beat of musical excerpts (that is, they will have a lower standard deviation of asynchrony than individuals who respond No to the target question) (H1). Self-report on a highly similar self-report question (‘I can tap in time with a musical beat’) with responses on a seven-point agreement Likert scale is also predicted to be correlated with tapping accuracy (H1a). Second, the target question will be associated with broader rhythm ability/engagement (measured with a rhythm scale from seven other self-report questions) (H2a), and beat synchronization task performance will reflect broader self-reported rhythm ability/engagement (H2b). Third, confidence (as either a personality trait or sureness in one’s own task performance) affects the reliability of self-reported beat synchronization (H3). Fourth, selected traits found to be genetically correlated with beat synchronization in the GWAS will be phenotypically correlated with beat synchronization task performance and the rhythm scale (H4). Specifically, better beat/rhythm is correlated with evening chronotype (H4a), less shortness of breath (H4b), more tinnitus and loud music exposure (H4c), and more smoking (H4d); and these associations will survive controlling for age, sex and education (H4e). Fifth, the responses to the target question will be positively correlated with musical engagement measured with the Gold-MSI (H5). Sixth, the associations in H4 will interact with being a musician, or more generally, with musical engagement (H6).

##### Participants

A total of *N* = 1,412 individuals met the participation criteria outlined in the preregistration (including passing the attention check item and not abandoning the study before completion). The study took place in Amazon Mechanical Turk, and all participants provided informed consent in accordance with the Max Planck Society Ethics Council’s approved protocol; the research complied with all relevant ethical regulations. The participants (728 females, 678 males and 6 who preferred not to answer) were 18–77 years old (mean, 36.3 years; s.d., 11.9) and had 1–2 years of self-reported musical experience (Table 1). To ensure that the tapping technology measured beat synchronization with high temporal fidelity, it was crucial that the participants complied with instructions to perform the tapping task (for example, using the laptop speakers instead of headphones, with minimal background noise and so on) and also used hardware and software without any technical issues that would preclude the recording signal (for example, malfunctioning speakers or microphones, or the use of strong noise cancellation technology; see ref. ^47^). Thus, several precautions, including calibration tests and practice trials, were taken to make sure the tapping technology would work effectively, excluding cases that did not meet the requirements (see Supplementary Methods and Results: section A for the details). A subset of *N* = 542 had appropriate hardware to complete all parts of the study (including the tapping tests), in line with power estimates for predetermined sample size (minimum *N* = 500 with usable tapping data) as detailed in the preregistration. Questionnaires were administered in the full sample of participants. The sample demographics are reported in Table 1. The demographics of the participants that completed the tapping experiment were highly similar to the full sample, as shown in the table; furthermore, 65.3% of the full sample and 64.9% of the tapping sample had a bachelor’s degree or higher. The participants were paid at a rate of approximately US$9 per hour depending on how much of the experiment they completed.

#### Data collection for Phenotype Experiment 2

The first questionnaire included self-report items, including the target question and also covering a variety of musical, health and interest phenotypes. The health phenotype questions were chosen from phenotypes (chronotype, smoking, shortness of breath and tinnitus) found to be genetically correlated with beat synchronization in our genetic analyses. The rhythm questions were selected for their particular relevance to various aspects of interacting/engaging with musical rhythm. The order of the questions was fixed for all participants. In addition, we used an attention check item^125^ between items 10 and 11 to exclude fraudulent responders, such as computer bots or disengaged participants responding randomly to the experiments. The end questionnaire consisted of items covering the following additional self-report topics: another question about being a musician, a task confidence rating question, a confidence scale, a 16-item short version of the Gold-MSI^43^ (the items were chosen due to their high reliability scores; reliability omega, 0.92) and a demographic questionnaire. The questionnaire items for Phenotype Experiment 2 are listed in Supplementary Methods and Results: section N.

##### Tapping technology

Beat synchronization is particularly challenging to study with online research, where variability in participants’ hardware and software can introduce delay in latency and jitter into the recorded time stamps^126,127^. Here we used REPP (see ref. ^47^ for the full details and a validation study of the technology), a robust cross-platform solution for measuring sensorimotor synchronization in online experiments that has high temporal fidelity and can work efficiently using hardware and software available to most participants online. To address core issues related to latency and jitter, REPP uses a free-field recording approach: specifically, the audio stimulus is played through the laptop speakers, and the original signal is simultaneously recorded with the participants’ tapping responses using the built-in microphone. The resulting recording is then analysed using signal processing techniques to extract and align timing cues with high temporal accuracy.

##### Beat synchronization task

The beat synchronization task procedure consisted of three parts: calibration tests, a practice phase and the main tapping phase. The participants started with the calibration tests, including a volume test to calibrate the volume of the laptop speakers to a level sufficient for detection by the microphone, a background noise test to make sure the participants were in a quiet environment and a tapping test to help the participants practise how to tap on the surface of their laptop in the right level and location to be detected by the microphone. The participants were then presented with the practice phase, which consisted of four 15 s trials of isochronous tapping to a metronome beat (two with an inter-onset interval of 500 ms and two with an inter-onset interval of 600 ms). Following the practice phase, the participants were presented with the main tapping task consisting of eight trials (four musical excerpts, each played twice), with each trial 30 s long. The order of presentation of the practice trials and test trials was randomized for each participant.

The musical excerpts were drawn from the MIREX 2006 Audio Beat Tracking database, in which musical excerpts had been annotated for beat locations by 30 listeners who tapped along to the music^128^. We chose four MIREX clips that represent different music genres with different tempos and tapping difficulty: track 1 (‘You’re the First, the Last, My Everything’ by Barry White), track 3 (‘El Contrapunto’ by Los Mensajeros de La Libertad), track 7 (‘Le Sacre du Printemps’ by Stravinsky) and track 19 (‘Possessed to Skate’ by Suicidal Tendencies) of the MIREX training set. On the basis of the annotations in ref. ^128^, we identified the target beat locations from those consistently produced by the annotators. Additional technical details are provided in the Supplementary Methods and Results: section A, and Supplementary Fig. 2 illustrates the instructions for the participants.

#### Data analysis

##### Beat synchronization task performance: tapping accuracy analysis

Let S_*t*_ and R_*t*_ be the stimulus and response onsets, respectively. In case of the metronome, S_*t*_ is the metronome onset (practice phase), and for the music clips, S_*t*_ is the target beat location based on the annotations. We define the asynchrony as a_*t*_ = R_*t*_ − S_*t*_. On the basis of prior work^129^, we chose the standard deviation of the asynchrony (s.d.(a_*t*_)) as our main target interest variable, as this appears to be a robust measure of individual performance and tightly linked to musical abilities^130^. We used metronome onsets to mark the beat metric level in an unambiguous way^131^. We emphasize that the metronome onsets were physically present only during the beginning and end of each clip. We used only the participant-produced asynchronies during the epoch at beats in which the guiding metronome was not present, in order to test the ability of the participants to synchronize to music without the metronome sounds (the results were nearly identical when we included all onsets, including the ones where physical metronome onsets were present). For the main test scores, we used the asynchronies computed relative to the virtual beat locations computed from prior human annotators in MIREX. We also computed vector length to confirm key associations of interest between the target question and beat synchronization accuracy (Supplementary Methods and Results: section A).

##### Regression analyses

In accordance with the Open Science Framework preregistration, we examined whether responses to self-reported beat synchronization phenotype were associated with objectively measured tapping accuracy and other self-reported measures of rhythm ability, confidence and musical sophistication using logistic regression and McFadden’s *R*^2^ (for H1, H2a, H3 and H5) and linear regression (for H1a and H2b). Likewise, we used linear regression to examine potential replication of cross-trait associations uncovered by genetic analyses (H4a–d) and to examine whether musical background interacted with the above associations (H6). The analyses were conducted in R v.3.5.1 (ref. ^132^). As described in our preregistration, individuals were recruited using Amazon Mechanical Turk and were included unless they failed an attention check item or abandoned the experiment before completing the study (*N* = 1,412). Usable tapping data were available for *N* = 542 individuals. The majority of exclusions were due to technical reasons detected by REPP’s signal processing pipeline during the practice trials (for example, poor signal, noisy environment, wearing headphones, issues with the laptop microphone or people not tapping at all), but some additional participants (*N* = 19) were excluded for not having enough usable trials during data analysis. Missing covariates were handled using pairwise deletion. All continuous measures met the assumption of normality (skew, ±2; kurtosis, ±4). The exclusion criteria are detailed in the Supplementary Methods and Results: section A.

### GWAS of beat synchronization

The GWAS summary statistics were generated from data acquired by personal genetics company 23andMe, Inc. Phenotypic status was based on responses to an English-language online questionnaire in which individuals self-reported ‘Yes’ (cases) or ‘No’ (controls) to the question ‘Can you clap in time with a musical beat?’. Individuals who responded ‘I’m not sure’ were excluded from the genomic dataset, as their data were not available. The GWAS included a total of 555,660 cases and 51,165 controls (total *N* = 606,825; mean age (s.d.), 52.09 (18.5); prevalence, 92%) who were unrelated individuals of European ancestry; the age range breakdown is provided in Table 1. Sample size for the GWAS was not predetermined, but recent GWASs of complex traits (for example, ref. ^133^) have indicated that very large sample sizes are needed to detect the genetic architecture of such traits. All individuals provided informed consent according to 23andMe’s human subject protocol, which is reviewed and approved by Ethical & Independent Review Services, a private institutional review board (http://www.eandireview.com); the study complied with all relevant ethical regulations.

The GWAS was conducted using logistic regression under an additive genetic model, while adjusting for age, sex, the top five principal components estimated from genetic data to control for population stratification, and indicators for genotype platforms to account for batch effects. We excluded SNPs with minor allele frequency < 0.01, low imputation quality (*R*^2^ < 0.3) and indels, resulting in 8,288,850 SNPs in the GWAS summary statistics. Genotyping and quality control details are provided in the Supplementary Methods and Results: section B.

### Post-GWAS enrichment analyses

#### FUMA-based analyses

The FUMA^58^ web application was used on the GWAS summary statistics to identify genomic loci along with the ‘sentinel’ SNPs that were independent in our analysis with a genome-wide significant *P* value (<5 × 10^−8^) that are in approximate LD with each other at *r*^2^ < 0.1 and to generate Manhattan plots and quantile–quantile plots. GWAS Catalogue associations for the top loci were performed in FUMA (Supplementary Table 16).

Next, using the GWAS summary statistics as input for MAGMA (v.1.08), we conducted a gene-based test of association, a gene property enrichment test and a gene-set enrichment analysis. Gene property analysis^56^ utilized GTEx v.8 data integrated in FUMA, with gene expression values log_2_-transformed and average transcript per million (TPM) per tissue type were adopted after winsorization at 50 based on GTEx RNA-seq data; this analysis was performed for 54 tissue types, where the result of gene analysis was tested for one side while conditioning on average expression across all tissue types. We also performed exploratory GSA^57^ in FUMA using 15,556 Gene Ontology gene sets from the MsigDB database^134,135^; a Bonferroni threshold of 3.2 × 10^−6^ was used.

#### SNP-based heritability and partitioned heritability

SNP-based heritability was computed with LDSC software^60^, and heritability estimates were adjusted to the liability scale on the basis of population prevalence of atypical beat synchronization of 3.0%–6.5% (Supplementary Table 3 and Supplementary Methods and Results: section C). We partitioned the heritability of beat synchronization by 52 broad functional categories (Supplementary Table 7) using stratified LDSC^60,63^ (Bonferroni-corrected significance level of *P* = 9.6 × 10^−4^). We hypothesized that SNPs falling into open chromatin regulatory regions (that is, accessible to transcriptional machinery) and regions with human-specific variation would be enriched for beat-synchronization-associated variation.

We further investigated (SNP-based) cell-type-specific and tissue-specific enrichments with LDSC–SEG^67^, using a total of 697 gene sets (3 Cahoy gene sets, 205 Multi-tissue gene expression sets and 489 Multi-tissue chromatin sets from the RoadMap Epigenomics and ENCODE datasets); the Bonferroni-corrected significance level for this analysis was 7.1 × 10^−5^ (Supplementary Table 8). The X chromosome was not included in these analyses or any subsequent analyses using LDSC, given that the file that is required for LDSC analysis (w_hm3_snplist) does not include chromosome X SNPs.

#### Evolutionary analyses

The set of HARs was taken from ref. ^65^. All variants in perfect LD (*r*^2^ = 1.0 in 1000 Genomes European participants) with variants in HARs were considered in the analysis. Similarly, variants tagging Neanderthal introgressed haplotypes were defined as in ref. ^136^. All variants in perfect LD with a Neanderthal tag SNP were considered Neanderthal variants. For each set, we performed stratified LDSC (v.1.0.0) with European LD scores and the baseline LD-score annotations v.2.1. The heritability enrichment is defined as the proportion of heritability explained by SNPs in the annotation divided by the proportion of SNPs in the annotation. Standard effect size (τ*), which quantifies the effects unique to the annotation, is the proportionate change in per-SNP heritability associated with a one-standard-deviation increase in the value of the annotation, conditional on other annotations in the baseline v.2.1 model^62^. To determine the expected number of overlaps between the *N* loci significantly associated with beat synchronization and HARs, we computed all overlaps between these sets of genomic regions (in hg19 coordinates) using bedtools2 (ref. ^137^). We then randomly shuffled the locations of HARs around the genome, choosing segments of equal lengths and avoiding gaps in the genome assembly. We repeated this process 10,000 times and for each iteration computed the number of overlaps observed with the significantly associated loci. On the basis of this empirical distribution created with no association between the region sets, we computed the enrichment and *P* value for the observed number of overlaps.

### Genetic correlations

The genetic correlation method is designed to show whether there is shared genetic variation linked to a particular trait (here, our beat synchronization trait) and traits measured in other GWAS studies. Given that beat synchronization is a brain-related trait with a motor periodicity component, the guiding principle of our selection criteria was foremost to explore its relationship with other brain-related traits as well as traits encompassing biological rhythms, and also leaving open the possibility of hitherto-unstudied relationships with general health. We thus curated GWAS summary statistics for 64 complex traits representing a broad range of phenotypic categories: cognitive, psychiatric, neuroimaging/other neurological, motor, other biological rhythms (circadian, heart and breathing), overall health/well-being and hearing (see Supplementary Table 10 and Supplementary Methods and Results: section G for the details of the source studies; the largest-sample and most up-to-date publicly available GWASs were used wherever possible). We estimated genetic correlations between beat synchronization and each of these traits using LDSC^72^, with a Bonferroni threshold of 7.5 × 10^−4^ (Supplementary Table 11).

### Beat synchronization PGS prediction of music engagement reported in health records

#### Overview

We examined whether beat synchronization PGSs would be associated with music engagement reported in health records. Individuals who disclosed music engagement to their care providers (which was subsequently recorded by their providers) were drawn from a recent phenome-wide study of 9,803 musicians^71^ identified from keyword searches of patient electronic health records in Vanderbilt University Medical Center’s de-identified research database (Synthetic Derivative). The phenotyping method was based on mining of clinical notes, using four keywords and 449 regular expressions (for example, ‘musician’ and ‘plays the piano’); see Supplementary Methods and Results: section F and ref. ^71^ for the details. The method was then validated with manually conducted chart review, with a positive predictive value of 93%. Here we accessed the subset of *N* = 1,259 musicians and 4,893 controls (matched for sex, median age (across the patients’ medical record), ethnicity, race and length of record) that were also part of the BioVU database and had genotyped data on file, to test the hypothesis that higher PGSs for beat synchronization would be associated with musical engagement operationalized as a having musician-related keywords or regular expressions recorded in an individual’s electronic health record.

We selected only individuals of European ancestry with genetic data that met standard quality control thresholds due to the poor performance of PGSs trained in individuals of one ancestry and applied to individuals of another. This resulted in *N* = 1,259 individuals (553 (44%) females; mean median age of record (s.d.), 53.1 (16.5)) as musician ‘cases’ and 4,893 controls (1,963 (40%) females; mean median age of record (s.d.), 53.2 (16.3)). See Supplementary Methods and Results: section F for details on the phenotyping, samples, genotyping and quality control.

#### PGSs

We used an identity by descent (IBD) filter of 0.2 to include only unrelated European samples of BioVU. PGSs were generated using the beat synchronization GWAS summary statistics, using software PRS_CS^138^. Briefly, this method uses a Bayesian regression framework and places a continuous shrinkage prior on SNP effect sizes; this method outperforms previous methods in terms of prediction accuracy, especially when the training sample size is large^138^, as is the case with the beat synchronization GWAS. The 1000 Genomes European reference set was used. The PGS was standardized to have a mean of 0 and s.d. of 1. Chromosome X was not included in the BioVU sample.

#### Data analysis

We conducted a logistic multivariable regression where the outcome variable was musician versus control, the predictor variable was PGS for beat synchronization and covariates included median age across the patient’s medical record, sex and the top ten principal components estimated from the BioVU genetic data.

### Reporting summary

Further information on research design is available in the Nature Research Reporting Summary linked to this article.

## Supplementary information

Supplementary Information Glossary and Supplementary Methods and Results, figures and references.

Reporting Summary

Supplementary Tables Supplementary Tables 1–19.

Supplementary Data 1 Results of gene property analysis on rhythm-related common factor GWAS (from Magma implemented in FUMA with GTex-v.8 54 tissue types) for Supplementary Fig. 9.

Supplementary Data 2 Source data including vector variables for Supplementary Fig. 3.

Supplementary Data 3 Source data LDSC–SEG results for Supplementary Figs. 5, 6 and 7.

## Data Availability

The full GWAS summary statistics for the 23andMe dataset will be made available through 23andMe to qualified researchers under an agreement that protects the privacy of the 23andMe participants. Please visit https://research.23andme.com/collaborate/#publication for more information and to apply to access the data. The top 10,000 SNPs of the GWAS and the data from the phenotype validation studies are available for reasonable research purposes from https://bitbucket.org/marianiarchou/beat-synchronization-gwas. Source data are provided with this paper.

## Source data

Source Data Fig. 1b Individual data used to plot the rhythm perception task results of phenotype validation study 1.
Source Data Fig. 1c–h Individual data used to plot the tapping synchronization task results of phenotype validation study 2.
Source Data Fig. 3a Results of the gene property analysis (from Magma implemented in FUMA with GTex-v.8 54 tissue types).
Source Data Fig. 3b LDSC–SEG heritability enrichment results.
Source Data Fig. 4 Genetic correlation results.
Source Data Fig. 5 Model output for genomic SEM results.

## Code availability

The code for the phenotype validation studies and the post-GWAS analyses is available at https://bitbucket.org/marianiarchou/beat-synchronization-gwas.
