# Supplementary material for: Genome-wide association study of musical beat synchronization demonstrates high polygenicity
Source: Nat Hum Behav. 2022 Jun 16;6(9):1292–309. doi: 10.1038/s41562-022-01359-x (PMC9489530; doi:10.1038/s41562-022-01359-x)
Supplement: Supplementary file 2 — Reporting Summary [file 41562_2022_1359_MOESM2_ESM.pdf]

nature research

Corresponding author(s): Reyna Gordon, Lea Davis & Maria Niarchou

Last updated by author(s): April 8, 2022

Reporting Summary

Nature Research wishes to improve the reproducibility of the work that we publish. This form provides structure for consistency and transparency in reporting. For further information on Nature Research policies, see our Editorial Policies and the Editorial Policy Checklist.

Statistics

For all statistical analyses, confirm that the following items are present in the figure legend, table legend, main text, or Methods section.

n/a	Confirmed	
☐	☒	The exact sample size (n) for each experimental group/condition, given as a discrete number and unit of measurement
☐	☒	A statement on whether measurements were taken from distinct samples or whether the same sample was measured repeatedly
☐	☒	The statistical test(s) used AND whether they are one- or two-sided<br>*Only common tests should be described solely by name; describe more complex techniques in the Methods section.*
☐	☒	A description of all covariates tested
☐	☒	A description of any assumptions or corrections, such as tests of normality and adjustment for multiple comparisons
☐	☒	A full description of the statistical parameters including central tendency (e.g. means) or other basic estimates (e.g. regression coefficient) AND variation (e.g. standard deviation) or associated estimates of uncertainty (e.g. confidence intervals)
☐	☒	For null hypothesis testing, the test statistic (e.g. F, t, r) with confidence intervals, effect sizes, degrees of freedom and P value noted<br>*Give P values as exact values whenever suitable.*
☒	☐	For Bayesian analysis, information on the choice of priors and Markov chain Monte Carlo settings
☒	☐	For hierarchical and complex designs, identification of the appropriate level for tests and full reporting of outcomes
☐	☒	Estimates of effect sizes (e.g. Cohen's d, Pearson's r), indicating how they were calculated

Our web collection on statistics for biologists contains articles on many of the points above.

Software and code

Policy information about availability of computer code

Data collection	23andMe, Inc. uses a custom pipeline for data collection.<br>The phenotype validation studies used Dallinger platform (https://github.com/Dallinger) in Amazon's Mechanical Turk portal for data collection, with custom scripts.
Data analysis	R (v1.2.1335 and 3.5.1), LD score regression (version 1.0.1), GCTA (v.1.92.1.beta6), PRS_CS, FUMA(v1.3.6a), MAGMA(as integrated by FUMA, v1.08), and GenomicSEM_0.0.2 . All above-mentioned software is publicly available. 23andMe, Inc. uses a custom pipeline for data analysis.

For manuscripts utilizing custom algorithms or software that are central to the research but not yet described in published literature, software must be made available to editors and reviewers. We strongly encourage code deposition in a community repository (e.g. GitHub). See the Nature Research guidelines for submitting code & software for further information.

Data

Policy information about availability of data

All manuscripts must include a data availability statement. This statement should provide the following information, where applicable:

- Accession codes, unique identifiers, or web links for publicly available datasets
- A list of figures that have associated raw data
- A description of any restrictions on data availability

The full GWAS summary statistics for the 23andMe dataset will be made available through 23andMe to qualified researchers under an agreement that protects the privacy of the 23andMe participants. Please visit research.23andme.com/collaborate/#publication for more information and to apply to access the data. The top 10,000 SNPs of the GWAS and the data from the phenotype validation studies are available for reasonable research purposes from https://bitbucket.org/marianiarchou/beat-synchronization-gwas.
The code for the phenotype validation studies and the post-GWAS analyses is available at https://bitbucket.org/marianiarchou/beat-synchronization-gwas.

Field-specific reporting

Please select the one below that is the best fit for your research. If you are not sure, read the appropriate sections before making your selection.

☒ Life sciences ☐ Behavioural & social sciences ☐ Ecological, evolutionary & environmental sciences

For a reference copy of the document with all sections, see nature.com/documents/nr-reporting-summary-flat.pdf

Life sciences study design

All studies must disclose on these points even when the disclosure is negative.

Sample size	No sample size for the GWAS was predetermined, but recent research on genetics of complex traits (for example, GWAS of IQ/educational attainment) has indicated that very large sample sizes are needed to detect the SNP-based heritability of such traits. To maximize power, we included the largest amount of data available from 23andMe on the musical rhythm phenotype (individuals of European ancestry participating in research who responded to the question, Can you clap in time with a musical beat?, referred to below as 'clap-beat question"). No exact sample size was pre-determined for phenotype validation experiment #1, but we estimated that between 600 and 1000 participants would participate across two waves of recruitment via Mechanical Turk, based on other ongoing studies. Phenotype validation experiment #2 was pre-registered (https://osf.io/exr2t) with specific recruitment criteria to obtain enough (at least N=500) usable data (given technical constraints) for the tapping portion of the study and having a larger sample (at least N=1000) for the questionnaire portions of the study (i.e. individuals who did not have the technical specifics to complete the tapping tasks were still kept in the study for the self-report questionnaires).
Data exclusions	In the GWAS, research participants who opted to respond to the clap-to-beat question and were unrelated and of European ancestry (see Online Methods) were included in the study; individuals who responded "I'm not sure" to the clap-beat question were not included.<br>In phenotype validation experiment #1, N=11 were excluded for incomplete items on the rhythm perception test (N=735 did the self-report questions and began the rhythm test; the N=724 in the final dataset had complete responses on both the self-report questions and the rhythm test). Regarding the phenotype validation experiment #2, participants were excluded for the following reasons: 1. Failing the first page of the attention check item in Part I (they were exclude during testing and did not advance to Part II).<br>2. Abandoning the experiment before completion of Part III. Participants who did not pass the technical calibration in the practice phase in Part II would advance directly to Part III, and their was used for self-report correlation analyses. All exclusion criteria were described in the pr-registration and are reported in the manuscript.
Replication	Our GWAS sample was not divided into a discover and replication sample in order to maximize power to detect genomic-signals of a musical rhythm phenotype. However, we were able to perform a proof-of-concept replication of the polygenic score for beat synchronization (derived from the GWAS) by applying it to individuals categorized as musicians in a healthcare biobank and compared to individuals with no mention of music-related keywords in their records, and found that polygenic score for beat synchronization significantly predicted musician status.<br><br>The data provided in the Supplementary tables, in addition to the availability of summary statistics to qualified researchers (via data transfer agreement), should be sufficient for other groups to attempt replication when musicality phenotypes become available in other large-scale genome-wide datasets.
Randomization	Assignment to groups was not random and was based on responses to the clap-beat question.
Blinding	Blinding was not used. Analysts were not blind to phenotypic status.

Reporting for specific materials, systems and methods

We require information from authors about some types of materials, experimental systems and methods used in many studies. Here, indicate whether each material, system or method listed is relevant to your study. If you are not sure if a list item applies to your research, read the appropriate section before selecting a response.

Materials & experimental systems

n/a	Involved in the study	
☒	☐	Antibodies
☒	☐	Eukaryotic cell lines
☒	☐	Palaeontology and archaeology
☒	☐	Animals and other organisms
☐	☒	Human research participants
☒	☐	Clinical data
☒	☐	Dual use research of concern

Methods

n/a	Involved in the study	
☒	☐	ChIP-seq
☒	☐	Flow cytometry
☒	☐	MRI-based neuroimaging

Human research participants

Policy information about studies involving human research participants

Population characteristics	The GWAS study sample was N=606,825 individuals of European ancestry (mean age 52 years, 59% females).<br>The Phenotype validation experiment #1 sample (N=724) had a mean age of 36 years and was 46% females.<br>The Phenotype validation experiment #2 sample (N=1,412) had a mean age of 36 years and was 52% females.
Recruitment	GWAS participants were 23andMe, Inc. customers who opted into research and answered the clap-beat question between 2015 and 2018. We do not believe that there is any self-selection bias that would differ these participants from participants who answered other questions in 23andMe's research questionnaires.<br>Both phenotype validation studies took place in Amazon's Mechanical Turk: the first in 2018 (phenotype validation experiment #1) and the second in 2020 (phenotype validation experiment #2). Participants were required to use the MTurk system. We recruited participants from the US (we used MTurk "US only" recruitment option) with HIT approval rate (percentage of approved task as reported by the MTurk system) of 95% or more.
Ethics oversight	23andMe's human subject protocol was reviewed and approved by Ethical & Independent Review Services, a private institutional review board.<br>Phenotype validation experiment #1 received ethical approval from the Columbia University Institutional Review board.<br>Phenotype validation experiment #2 received ethical approval from the Max Planck Society Ethics Council (approved protocol (application 2018_38).

Note that full information on the approval of the study protocol must also be provided in the manuscript.
