# Supplementary material for: Genome-wide association study of musical beat synchronization demonstrates high polygenicity
Source: Nat Hum Behav. 2022 Jun 16;6(9):1292–309. doi: 10.1038/s41562-022-01359-x (PMC9489530; doi:10.1038/s41562-022-01359-x)
Supplement: Source Data Fig. 5 — Model output for genomic SEM results. [file 41562_2022_1359_MOESM12_ESM.docx]

**Model Output for Figure 5**

*Genomic SEM model of clapbeat, speed, grip strength, walk pace, & peak expiratory flow*

*Highlighted values are displayed in the figure (95% CIs based on corresponding standard errors)*

*_________________________________________________________________________________________*

Model2 <- 'F1 =~ NA*expiratory + gripRight + RT + clapbeat + walkPace

+ F1 ~~ 1*F1

+ walkPace ~~ gripRight

+ '

> Model2_output <- usermodel(LDSCoutput_W, model = Model2)

[1] "Running primary model"

[1] "Calculating CFI"

[1] "Calculating Standardized Results"

[1] "Calculating SRMR"

elapsed

0.56

> Model2_output

$modelfit

chisq df p_chisq AIC CFI SRMR

df 10.84615 4 0.02834857 32.84615 0 .9834456 0.01688696

$results

lhs op rhs Unstand_Est Unstand_SE STD_Genotype STD_Genotype_SE STD_All p_value

5 F1 =~ expiratory 0.12732712 0.0109035070013261 0.3462264 0.0296486785090109 0.3462264 1.658565e-31

6 F1 =~ gripRight 0.16782225 0.0137730468807364 0.4983651 0.0409004485162103 0.4983651 3.744280e-34

7 F1 =~ RT 0.09534035 0.0101937247675904 0.3497178 0.0373915747211739 0.3497178 8.531956e-21

4 F1 =~ clapbeat 0.13815240 0.0144057457139605 0.3379820 0.0352428208838132 0.3379820 8.800802e-22

8 F1 =~ walkPace 0.08405546 0.0117898003472843 0.3131744 0.0439265218274036 0.3131744 1.007291e-12

10 gripRight ~~ walkPace 0.01147500 0.00283735409538393 0.1269617 0.031392947598701 0.1542004 5.248850e-05

2 expiratory ~~ expiratory 0.11903299 0.00759770081454443 0.8801272 0.0561772434059124 0.8801273 2.544041e-55

9 gripRight ~~ gripRight 0.08523313 0.00558054623737376 0.7516322 0.0492122325584034 0.7516322 1.152649e-52

11 RT ~~ RT 0.06523239 0.00341748476596556 0.8776975 0.045982042841531 0.8776975 3.186944e-81

1 clapbeat ~~ clapbeat 0.14799614 0.00635832819708419 0.8857682 0.0380550908818621 0.8857682 7.769437e-120

12 walkPace ~~ walkPace 0.06497222 0.00275540721377133 0.9019218 0.0382495859483822 0.9019218 6.197912e-123

3 F1 ~~ F1 1.00000000 1.0000000 1.0000000 NA
